# Applications and advances of multi-omics technologies in gastrointestinal tumors

**DOI:** 10.3389/fmed.2025.1630788

**Published:** 2025-07-23

**Authors:** Yuqing Liu, Feng Gao, Yang Cheng, Liang Qi, Haining Yu

**Affiliations:** Medical Equipment Department, Shandong Cancer Hospital and Institute, Shandong First Medical University and Shandong Academy of Medical Sciences, Jinan, Shandong, China

**Keywords:** gastrointestinal tumors, multi-omics technologies, single-cell genomics, early screening, biomarkers, treatment optimization

## Abstract

Gastrointestinal tumors pose a significant clinical challenge due to their high heterogeneity and the difficulties in early diagnosis. The article systematically reviews the latest advances in multi-omics technologies in gastrointestinal tumor research, focusing on their contributions to early screening, biomarker discovery, and treatment optimization. Genomics reveals genetic characteristics and heterogeneity of tumors; transcriptomics helps identify molecular subtypes and potential therapeutic targets; proteomics provides important information on core proteins and the immune microenvironment; and metabolomics offers promising biomarkers for early diagnosis. Furthermore, emerging fields such as epigenomics, metagenomics, and lipidomics, through the construction of multi-scale frameworks, have opened new paths for molecular subtyping and targeted therapy. By integrating these multi-dimensional data, multi-omics integration enables a panoramic dissection of driver mutations, dynamic signaling pathways, and metabolic-immune interactions. However, challenges such as data heterogeneity, insufficient algorithm generalization, and high costs limit clinical translation. In the future, the integration of single-cell multi-omics, artificial intelligence, and deep learning technologies with multi-omics may offer more efficient strategies for the precise diagnosis and personalized treatment of gastrointestinal tumors.

## 1 Introduction

Gastrointestinal tumors, including gastric cancer (GC), colorectal cancer (CRC), and esophageal cancer, are among the leading causes of cancer-related mortality worldwide, with over 5 million new cases and nearly 3.5 million deaths annually, accounting for more than 25% of the global cancer burden (1). Despite progress in conventional treatments such as surgery, chemotherapy, and targeted therapies, patient prognosis remains limited by two core challenges: tumor heterogeneity and the lack of reliable early diagnostic biomarkers (2).

Tumor heterogeneity manifests as spatiotemporal dynamics in molecular features. For instance, approximately 50% of GC patients are diagnosed at advanced stages, and the 5-year survival rate for metastatic CRC is below 15% (3). This clinical dilemma stems from the interplay of multilayered biological processes, including genomic instability, epigenetic dysregulation, metabolic reprogramming, and immune microenvironment remodeling (4–6). Traditional single-omics studies (e.g., genomics or proteomics) can reveal changes at specific molecular levels but struggle to elucidate the synergistic mechanisms driving tumor initiation and progression through multi-omics interaction networks (7, 8). For example, KRAS mutations require transcriptomic analysis to uncover their regulatory effects on the MAPK/ERK pathway (9, 10), while metabolomics can further clarify mutation-induced metabolic phenotypes such as the Warburg effect (11, 12).

The integrated application of multi-omics technologies offers systemic solutions to address critical bottlenecks in gastrointestinal tumor research (13, 14). By harmonizing multi-dimensional data from genomics, transcriptomics, proteomics, and metabolomics, integrated multi-omics reveals driver mutations, signaling pathways, and metabolic-immune crosstalk, offering systemic insights (15, 16). For example, in CRC, whole-exome sequencing (WES) revealed that APC gene deletion activates the Wnt/β-catenin pathway (17), while metabolomics further demonstrated that this pathway drives glutamine metabolic reprogramming through the upregulation of glutamine synthetase (18). Such cross-omics integration not only overcomes the limitations of single-marker analyses (e.g., HER2 protein overexpression requiring validation by ERBB2 gene amplification) but also enables dynamic tracking of therapeutic resistance through liquid biopsy multi-omics (e.g., ctDNA mutations combined with exosomal PD-L1 protein) (19). For instance, in metastatic CRC, combined detection of KRAS G12D mutations and exosomal EGFR phosphorylation levels predicts cetuximab resistance 12 weeks in advance (20).

In recent years, the deep integration of artificial intelligence (AI) with multi-omics has revolutionized precision medicine (21, 22). Machine learning algorithms, such as deep residual networks (ResNet-101), analyze heterogeneous multi-omics datasets to identify potential biomarkers and construct prognostic models (23, 24). For instance, the deep residual network (ResNet-101) integrated multi-omics data from CRC to build an mass spectrometry imaging (MSI) status prediction model, achieving an AUC of 0.93 (95% CI: 0.917–0.948) in 10,452 samples, and maintaining an AUC of 0.89 (95% CI: 0.866–0.914) in an independent external validation cohort, significantly outperforming traditional PCR testing (AUC = 0.85) (25). Single-cell spatial multi-omics technologies [e.g., single-cell RNA sequencing (scRNA-seq) combined with desorption electrospray ionization mass spectrometry imaging (DESI-MSI)] dissect cellular heterogeneity and metabolic-immune interaction networks within the tumor microenvironment (26). These approaches have uncovered metabolic-immunoregulatory features of cancer stem cell subpopulations, such as CD133+ cells secreting IL-6 (>35 pg/ml) to polarize M2 macrophages (CD206+ proportion increased from 12% to 54%, *p* < 0.001) and suppress CD8+ T cell infiltration via spatial lactate gradients (>5 mmol/mm^2^) (27, 28). These discoveries provide novel pathways for developing CAR-T therapies (e.g., dual-targeting CD133/IL-6R) and metabolic intervention strategies (e.g., LDHA inhibitors) (29, 30).

This essay attempts to methodically review the development of multi-omics technologies in gastrointestinal tumor research, with a focus on their functions in early screening, biomarker discovery, and treatment optimization, as well as the use of methodological references for the clinical translation of multi-omics-based precision diagnostic and therapeutic strategies.

## 2 Multi-omics technologies

Multi-omics technologies refer to the integrated analysis of data from multiple omics levels, encompassing genomics, transcriptomics, proteomics, metabolomics, epigenomics, and metagenomics. Each omics technology has distinct characteristics and applications, as shown in Table 1. This article primarily explores the applications of multi-omics technologies in gastrointestinal tumors.

**TABLE 1:** Comparison of omics technologies (technical methods, application examples, advantages, and challenges).

Omics technology	Technical methods	Application examples	Advantages	Challenges
Genomics	Targeted sequencing (panel sequencing, amplicon sequencing) (33)	Cancer driver gene screening, genetic disease diagnosis	Cost-effective, covers specific gene regions	Relies on prior knowledge, may miss novel variants
	High-throughput sequencing (WGS, WES) (34, 35)	Rare disease whole-genome analysis, evolutionary studies	Comprehensive detection of SNV/CNV/structural variants	Large data volume, high cost
	Liquid biopsy (ctDNA, CTC isolation technology) (40)	Early tumor screening, treatment resistance monitoring	Non-invasive sampling, dynamic monitoring	Sensitivity limited by low-frequency mutations
	Third-generation sequencing (e.g., PacBio, Oxford Nanopore) (37, 38)	Developmental epigenetic regulation studies, tumor methylation profiling	Single-base resolution	High DNA input requirement, complex data analysis
Transcriptomics	RNA-seq (strand-specific-library construction, single-cell RNA-seq) (47, 94)	Differential expression analysis, alternative splicing studies	Comprehensive transcriptome coverage, accurate quantification	Short read length limits isoform detection
	Spatial transcriptomics (10x Visium, Slide-seq) (112, 115)	Tumor microenvironment analysis, brain region-specific expression	Preserves spatial location information	Limited resolution (55 μm), high cost
Proteomics	LC-MS/MS (DIA/DDA modes, phosphorylation/glycosylation enrichment) (144, 145)	Phosphorylation signaling pathway studies, biomarker screening	High sensitivity, identifies PTMs	Limited dynamic range, requires pre-fractionation
	Mass spectrometry imaging (MALDI-TOF, DESI) (146)	Tumor tissue heterogeneity analysis	Visualizes spatial distribution	Lower resolution (50–200 μm)
	Single-cell proteomics (SCoPE-MS, CyTOF) (61)	Tumor stem cell identification	Overcomes bulk sample limitations	Immature technology, sparse data
	Protein interactomics (Co-IP/MS, AP-MS) (147)	Signal network construction	Reveals functional complexes	High false-positive rate
Metabolomics	Single-cell metabolomics (AFADESI, LA-ICP-MS) (148)	Tumor metabolic heterogeneity studies	Single-cell resolution	Difficult metabolite identification
	Mass spectrometry (GC-MS, CE-MS) (79)	Metabolic disease classification, drug toxicity assessment	Broad-spectrum small molecule detection	Requires derivatization
	Nuclear magnetic resonance (1H-NMR, 13C-NMR) (78)	Urine metabolic profiling	Non-destructive detection, accurate quantification	Lower sensitivity
Epigenomics	Single-cell epigenomics (scATAC-seq, scCOOL-seq) (127)	Tumor cell chromatin accessibility analysis	Single-cell analysis, reveals gene regulation	Large data volume, technical complexity
Metagenomics	Spatial metagenomics (GeoMx DSP) (5)	Microbial community analysis	Efficient, culture-free, covers most microbial types	Lacks functional information, amplification bias
Lipidomics	Liquid chromatography-mass spectrometry (LC-MS) (138)	Lipid metabolism abnormality analysis	High sensitivity, separates complex lipids	Complex sample preparation, cumbersome data analysis
Matrixomics	Single-cell matrixomics (DBiT-seq) (140)	Biological tissue lipid distribution analysis	High resolution, non-invasive analysis	Limited spatial resolution, difficult data interpretation

### 2.1 Core omics technologies

**FIGURE 1:**
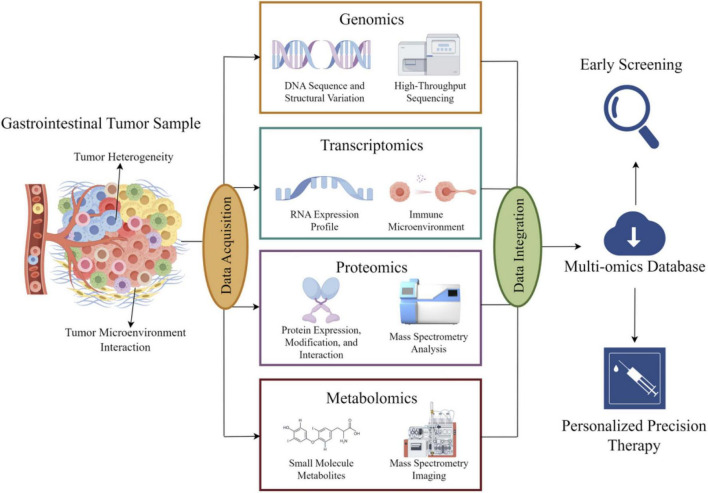
Schematic diagram of the integrated application of basic omics technologies in digestive tract tumors: information extracted from digestive tract tumors is processed using genomics, transcriptomics, proteomics, and metabolomics methods. The processed information is aggregated, analyzed, and uploaded to cloud storage, providing data support for early diagnosis and precision treatment of tumors.

Core omics technologies, including genomics, transcriptomics, metabolomics, and proteomics, form the foundational pillars of multi-omics research. The schematic illustration of multi-omics integration in gastrointestinal cancers is shown in Figure 1, providing a comprehensive and systematic perspective for elucidating the molecular mechanisms of gastrointestinal tumors (31).

#### 2.1.1 Genomics

Genomics, through detailed analysis of DNA sequences and structural changes in gastrointestinal tumors [e.g., whole-genome sequencing (WGS) and WES], reveals the correlation between tumor heterogeneity and genetic complexity: the higher the tumor heterogeneity, the greater its genetic complexity. This characteristic provides a foundation for elucidating the molecular mechanisms of tumorigenesis (32). Targeted sequencing panels enables precise identification of high-frequency gene abnormalities (e.g., KRAS and TP53), guiding clinical treatment decisions for CRC, particularly in the field of immunology (33). High-throughput sequencing technologies, such as WGS and WES, identify critical gene abnormalities and aberrant signaling pathways (34, 35). For instance, TP53, KRAS, and BRAF are prevalent in gastric, colorectal, and esophageal cancers, closely associated with genomic instability and the RAS-MAPK signaling pathway, respectively (36). These findings broaden our understanding of carcinogenesis and provide a cornerstone for personalized treatment and molecular subtyping.

Recent advances in genomics owe much to innovations in sequencing technologies. Third-generation sequencing platforms (e.g., PacBio and Oxford Nanopore) can detect long DNA fragments and complex structural variations (e.g., large insertions/deletions and chromosomal rearrangements), addressing limitations of short-read sequencing in detecting complex genomic rearrangements (37, 38). These technologies have uncovered previously underrecognized features of genomic instability in gastrointestinal tumors, such as the role of chromothripsis in esophageal cancer (39). Additionally, liquid biopsy techniques using circulating tumor DNA (ctDNA) capture tumor-derived DNA fragments in blood, offering non-invasive approaches for early screening and dynamic monitoring (40). For example, detection of specific mutations (e.g., KRAS G12D) in ctDNA has been applied to early diagnosis and recurrence risk assessment in CRC, with continually improving sensitivity and specificity (41).

However, genomics in gastrointestinal tumor research faces challenges. Tumor sample purity is often compromised by contamination from normal cells, complicating the detection of low-frequency mutations (42). Moreover, interpreting genomic data relies on functional annotation databases, which remain incomplete for non-coding region variants and complex rearrangements (43). In the future, integrating AI and big data analytics is expected to address these challenges by developing more accurate mutation function prediction models, advancing the clinical translation of genomics (44).

#### 2.1.2 Transcriptomics

Transcriptomics gives a unique approach for studying the dynamic molecular characteristics of gastrointestinal tumors by evaluating RNA expression profiles and regulatory networks (45). Unlike genomes, which focuses on static DNA variations, transcriptomics capture dynamic changes in gene expression, revealing complicated interactions between tumor cells and their microenvironment (46). RNA sequencing (RNA-seq), the principal transcriptomics technology, comprehensively detects expression levels of mRNA, lncRNA, and microRNA, systematically mapping gene expression profiles in gastrointestinal tumors such gastric and CRC. This has indicated abnormal activation patterns of important signaling pathways (e.g., TGF-β and PI3K-Akt) (47, 48). For example, in CRC, overexpression of WNT pathway target genes (e.g., MYC and AXIN2) is strongly linked to the adenoma-carcinoma sequence progression (49), while high Claudin 18.2 expression in GC has developed as a target for antibody-drug conjugate development (50).

Transcriptomics is a key component of tumor immune microenvironment research is transcriptomics. Researchers can describe the structure and functional status of immune cell subsets (e.g., T cells and macrophages) by examining the expression of RNA in tumor tissues (51). For example, in esophageal cancer, high PD-L1 mRNA expression frequently suggests an immunosuppressive microenvironment, whereas CD8+ T cell-related gene expression is correlated with immunotherapy response (52). To predict patient responses to checkpoint inhibitors, transcriptomics-based immune scoring systems (e.g., CIBERSORT) have been used to support precision immunotherapy (53). Additionally, tumor-associated fibroblasts (CAF) and matrix remodeling are linked to gene expression patterns found in transcriptomics, which are strongly associated with tumor invasion and metastasis. For instance, the TGF-β signaling pathway is frequently activated in GC by the high expression of CAF markers (e.g., FAP and ACTA2), which suggests matrix remodeling as a possible therapeutic target (54).

Another significant advance in transcriptomics is its application in studying fusion genes and alternative splicing. While fusion genes (e.g., EML4-ALK) are relatively rare in gastrointestinal tumors, specific subtypes (e.g., NTRK fusions in CRC) offer opportunities for targeted therapy (55). Alternative splicing events generate functionally distinct protein isoforms, increasing tumor heterogeneity and adaptability. For instance, alternative splicing variants of TP53 in GC have been associated with chemotherapy resistance (56).

Despite the immense potential of transcriptomics in biomarker discovery, batch effects, RNA degradation, and the reproducibility of dynamic expression profiles remain challenges for clinical applications (57). In the future, real-time RNA analysis based on nanopore sequencing, multiplex fluorescence *in situ* hybridization (mFISH), and AI-driven expression pattern recognition (e.g., deep learning models for predicting chemotherapy sensitivity) will emerge as novel solutions (58, 59).

#### 2.1.3 Proteomics

Proteomics focuses on comprehensively analyzing protein expression, modifications, and interactions, directly reflecting the terminal effects of gene expression and tumor functional phenotypes (60). With breakthroughs in mass spectrometry (MS) technologies (e.g., Orbitrap and TimTOF platforms), proteomics has evolved from “qualitative description” to “quantitative precision medicine.” In gastrointestinal tumors, proteomics not only elucidates effector proteins of driver mutations but also identifies key targets for therapy resistance (61, 62).

Proteomics play a vital role in the study of digestive tract tumors, particularly in uncovering key driver proteins and their associated signaling pathways. For example, in GC, MS has identified unusually high expression and phosphorylation levels of receptor tyrosine kinases such as HER2 and EGFR. These discoveries have been instrumental in driving the development of targeted therapies, such as trastuzumab (63). In CRC, proteomic analysis has demonstrated that downstream signaling molecules linked to KRAS mutations—such as proteins in the MAPK and PI3K-AKT pathways—show varying expression across different tumor subtypes, suggesting they could serve as promising new therapeutic targets (64). Moreover, proteomics is a powerful tool in exploring the tumor immune microenvironment. By examining the protein expression on the surface of tumor-associated macrophages (TAMs) and T cells, researchers can gain insights into mechanisms of immune suppression, such as the interaction between PD-L1 and PD-1, providing a scientific foundation for enhancing the effectiveness of immune checkpoint inhibitors (65, 66).

Proteomics exhibits remarkable advantages in the discovery of biomarkers. The early screening of gastrointestinal tumors relies on markers with high specificity and sensitivity. By examining the protein profiles in serum, saliva, and tissue exudates, proteomics can pinpoint specific proteins linked to tumor development (67). For instance, in GC studies, MS has revealed alterations in the pepsinogen (PGI/II) ratio in serum, which could serve as a potential indicator for early diagnosis (68). Moreover, proteomics can uncover dynamic changes in proteins associated with drug resistance by comparing protein profiles before and after treatment. For example, in research involving oxaliplatin-resistant CRC patients, proteomics has identified the overexpression of certain ABC transporter proteins, offering valuable insights for designing combination therapy strategies (69).

Despite significant progress, proteomics faces challenges such as complex sample preprocessing (e.g., difficulties in enriching low-abundance proteins) and limited big data analysis capabilities (70). To address this, AI-driven integration of proteomics data has emerged as a trend. For example, deep learning-based protein interaction network prediction models (such as DeepInteract) integrate GC-related functional genomics data (e.g., differentially expressed genes, survival analysis, and pathway enrichment analysis) to perform network topology analysis and key node identification. These deep learning architectures [typically based on graph neural networks (GNN) or transformers] can automatically learn latent patterns and feature representations of protein interactions from complex datasets, particularly for weak or transient interactions that are difficult to capture experimentally. This approach has been successfully applied to target screening in GC, significantly improving the discovery efficiency of membrane proteins (e.g., B7-H6) (71, 72). In the future, the integration of proteomics with spatial multi-omics will further reveal the molecular landscape of tumor-microenvironment interactions, advancing personalized treatment strategies (73).

#### 2.1.4 Metabolomics

Metabolomics, a key branch of systems biology, focuses on the global analysis of small-molecule metabolites (e.g., amino acids, lipids, and carbohydrates) in biological systems, revealing dynamic changes in metabolic networks and their associations with disease phenotypes through high-throughput technologies (74). Compared to genomics and transcriptomics, metabolomics is closer to the phenotypic endpoint, directly reflecting metabolic reprogramming in tumor cells and their interactions with the microenvironment (75). In gastrointestinal tumor research, the core value of metabolomics lies in deciphering tumor-specific metabolic abnormalities, providing critical clues for early diagnosis, molecular subtyping, and therapeutic target discovery (76).

The continuous advancements in MS and nuclear magnetic resonance (NMR) technologies have significantly enhanced the sensitivity and resolution of metabolomics (77, 78). For instance, liquid chromatography-mass spectrometry (LC-MS) is now capable of precisely detecting low-abundance metabolites (79), while high-resolution mass spectrometry (HRMS) excels at resolving thousands of metabolites in complex biological samples (80). In CRC research, metabolic profiling has revealed distinct differences in metabolites between tumor and normal tissues (81). Specifically, the abnormal accumulation of tricarboxylic acid (TCA) cycle intermediates and the dysregulation of lipid metabolism stand out prominently, suggesting that mitochondrial dysfunction may be a critical driver of metabolic heterogeneity in CRC. Furthermore, spatial metabolomics provides a novel perspective for exploring the spatial heterogeneity of the tumor microenvironment. By integrating MSI technology, researchers can directly map the distribution of metabolites on tissue sections, thereby uncovering differences in metabolic gradients between the tumor periphery and core regions (82). For example, in GC research, spatial metabolomics has demonstrated a marked enhancement of glutamine metabolism at the tumor invasive front, a phenomenon closely linked to the formation of an immunosuppressive microenvironment (83).

Several different biomarkers for gastrointestinal (GI) tumors were successfully identified through metabolomics research (84). For example, a targeted metabolomics study conducted in the field of GC evaluated the results of 702 plasma samples and found that metabolites such as alanine and glutamate in urine have diagnostic value, with a specificity of 88.03% for the samples, which exceeded the results of standard serum markers such as CEA and CA19-9 (85). Metabolomics can also be used to determine treatment response; multivariate integration analysis showed that decreased lactate levels and reduced glycolysis after apatinib treatment were associated with metabolic reprogramming in HER2-positive GC, which suggests that this method can be used to predict therapeutic efficacy (86).

The problems faced by metabolomics include complex data integration and a lack of standardization. The dynamic range of metabolites can span 9 orders of magnitude, with significant differences between batches. Additionally, the high degree of interconnection of metabolic pathways limits the reliability of single-metabolite markers (87). To address this issue, multi-omics integration techniques (e.g., combined metabolomics-proteomics analysis) can be used to construct comprehensive metabolic regulatory networks (88). For example, in liver cancer, the accumulation of L-glutamine is associated with the activation of the mTOR pathway and the inhibition of autophagy, and drugs targeting this pathway can reverse metabolic abnormalities and inhibit tumor growth (89).

### 2.2 Single-cell and spatial omics

In recent years, breakthrough advancements in single-cell omics and spatial omics technologies have opened up entirely new dimensions for digestive tract tumor research (90). Single-cell omics technologies, such as scRNA-seq and single-cell ATAC sequencing (scATAC-seq), systematically reveal the heterogeneity and dynamic evolutionary patterns of tumor cell subpopulations by resolving the genomic, transcriptomic, or epigenetic features of individual cells within tumors at high resolution (91). Spatial omics technologies, such as 10x Visium and Multiplexed Error-Robust Fluorescence *In Situ* Hybridization (MERFISH), enable three-dimensional localization and interaction network analysis of immune cells, stromal cells, and metabolites within the tumor microenvironment by preserving *in situ* spatial information of tissues (92). The integration of both approaches, exemplified by frameworks like SC-SpaceOmic, not only elucidates the spatiotemporal dynamics linking tumor heterogeneity and microenvironment remodeling at the molecular level but also provides precision tools for clinical translation (93).

#### 2.2.1 Single-cell omics

Single-cell omics technologies overcome the limitations of traditional omics approaches, which lose cellular heterogeneity due to sample homogenization, by analyzing the genome, transcriptome, epigenome, or proteome of individual cells (94). First, single-cell omics has shown immense potential in resolving tumor cell heterogeneity. Digestive tract tumors typically consist of multiple subclones with significant differences in genetic mutations, gene expression, and epigenetic regulation (95). For instance, scRNA-seq has been widely applied in gastric and CRC research, enabling researchers to identify distinct tumor cell subpopulations and their molecular characteristics through transcriptomic analysis of thousands of cells in tumor tissues (95). Additionally, single-cell genomics can detect rare tumor cell populations, such as circulating tumor cells (CTCs) or drug-resistant subpopulations, which play critical roles in tumor recurrence and treatment resistance (96).

Single-cell omics technologies have provided revolutionary tools for deeply analyzing the cellular heterogeneity and functional regulatory networks within the digestive tract tumor microenvironment. The digestive tract tumor microenvironment encompasses highly complex cellular populations, such as cytotoxic T lymphocytes (CD8+ T cells) with immune surveillance functions, B cells involved in antigen presentation, TAMs with highly plastic phenotypes, and cancer-associated fibroblasts (CAFs) that regulate invasion and metastasis through the secretion of extracellular matrix (ECM) components (97, 98). Traditional omics methods, limited by their resolution, struggle to precisely distinguish the molecular characteristics and interaction relationships among these cell subpopulations. In contrast, scRNA-seq, by analyzing the gene expression profiles of individual cells, can systematically reveal their functional state differentiation trajectories (99). For example, in esophageal squamous cell carcinoma, recent studies utilizing scRNA-seq combined with flow cytometry have confirmed a dynamic imbalance between M1-type (pro-inflammatory) and M2-type (immunosuppressive) polarization states of TAMs within the tumor microenvironment. Specifically, M2-type TAMs highly express CD206 and IL-10, which are significantly associated with shortened overall survival in patients (100). Furthermore, single-cell multi-omics analyses have uncovered the molecular regulatory mechanisms underlying T cell exhaustion: in the GC microenvironment, terminally exhausted CD8+ T cells not only persistently overexpress immune checkpoint molecules such as PD-1 and CTLA-4, but their epigenetic modifications (e.g., DNA methylation silencing the IFN-γ locus) have also been shown to reversibly regulate chemotherapy resistance (101, 102). These discoveries not only provide a theoretical basis for combination treatment strategies targeting the tumor microenvironment (such as PD-1 inhibitors combined with epigenetic drugs) but also promote the design of clinical trials for personalized immunotherapy based on single-cell subtyping.

Single-cell omics technology provides a unique research perspective for analyzing the spatiotemporal evolution patterns of digestive tract tumors. The dynamic process of tumor development involves multi-level synergistic effects of genetic variations (such as driver gene mutations) and epigenetic regulations (such as changes in chromatin accessibility) (103). Integrative multi-omics at single-cell resolution can precisely dissect the accumulation of such events and the mechanisms of clonal selection (104). For instance, in CRC, the expansion of KRAS G12D mutant subclones is not only directly related to resistance to anti-EGFR therapy, but its epigenetic features (such as the opening of AP-1 transcription factor binding sites) can also induce a pro-metastatic phenotype in stromal cells by activating IL-11 paracrine signaling (105). These findings provide experimental evidence for intervention strategies targeting key nodes in clonal evolution, such as the combination of KRAS inhibitors and metabolic reprogramming modulators (106).

Despite significant improvement, single-cell omics programs still have problems such as data sparsity, batch effects, and the complexity of multi-omics integration (107). In recent years, deep learning-based data imputation algorithms (e.g., scGNN) and cross-modal alignment tools (e.g., scCross) have considerably increased the integration efficiency of single-cell multi-omics data (108, 109). Also, the integration of single-cell multi-omics with organoid models (e.g., patient-derived gastric cancer organoids) has offered new paradigms for *in vitro* simulation of tumor evolution and drug screening (110).

#### 2.2.2 Spatial omics

Spatial omics preserves the spatial location information of cells within tissues, enabling the three-dimensional correlation of molecular characteristics with tissue architecture, thus addressing the limitation of single-cell sequencing technologies that lose spatial context (111).

Spatial transcriptomics technology, by integrating *in situ* gene expression with spatial coordinate information, provides a critical tool for dissecting the microenvironment heterogeneity of gastrointestinal tumors. Taking the widely used 10x Visium platform as an example, its core technology relies on spatial barcoding capture: fresh frozen or FFPE tissue sections are placed on slides covered with millions of unique oligonucleotide probes (containing spatial barcodes and poly (dT) capture sequences). After tissue permeabilization, released mRNAs are captured by adjacent probes, enabling *in situ* reverse transcription and the construction of barcoded cDNA libraries. Through high-throughput sequencing, gene expression data can be precisely mapped to the two-dimensional coordinates of the tissue section, achieving whole-transcriptome analysis while preserving the *in situ* spatial localization of cells. However, its spatial resolution limits the ability to precisely resolve spatial heterogeneity and fine cellular interactions at the single-cell level (112). To address this, MERFISH, a technique with ultra-high spatial resolution (∼100–200 nm, subcellular level) and single-molecule detection sensitivity, can effectively complement Visium’s shortcomings. MERFISH employs sophisticated barcode designs and multiple rounds of imaging to simultaneously detect hundreds to thousands of genes (typically ranging from 100 to over 10,000 genes) in a single tissue section, accurately mapping the subcellular localization of each RNA molecule within the tissue. By integrating these two technologies—Visium providing a whole-transcriptome view and MERFISH offering high-resolution targeted information—researchers can further dissect spatial transcriptomic features and complex intercellular interactions at the single-cell level while preserving the tissue’s spatial context (113).

In GC research, spatial transcriptomics has revealed significant tumor regional heterogeneity: cells at the tumor periphery highly express epithelial-mesenchymal transition (EMT)-related genes, while cells in the core exhibit stronger proliferation and metabolic activity. Spatial metabolomics further corroborates and expands on these findings at the metabolic level, showing substantial lactate accumulation in the core region, whereas fatty acid oxidation metabolism predominates at the periphery (114). These spatially distinct transcriptional and metabolic patterns provide a critical foundation for understanding tumor progression mechanisms and developing targeted therapies, particularly metabolism-targeted drugs. Additionally, spatial transcriptomics has revolutionized the study of the spatial topology of the tumor immune microenvironment. For instance, in microsatellite instability-high (MSI-H) CRC, three-dimensional reconstruction models from Visium data show that activated (GZMB+, and IFN-γ+) CD8+ T cells tend to cluster at the tumor-stroma interface, forming tight spatial interaction clusters (average distance <20 μm) with CXCL9-expressing myeloid cells. Notably, the density of these interaction clusters is significantly positively correlated with patient response rates to PD-1 inhibitor therapy (*r* = 0.67, *p* = 0.008) (115). Targeted delivery strategies based on this finding, such as local injection of liposome-encapsulated CXCL9 mRNA, have been shown in organoid models to significantly enhance T cell infiltration efficiency by 4.3-fold (*p* < 0.01), offering a spatially precise intervention pathway for optimizing immunotherapy in solid tumors (116). Furthermore, spatial proteomics, utilizing techniques such as multiplexed immunofluorescence or MSI, is increasingly widely applied. For example, in esophageal cancer research, it has revealed spatial heterogeneity of metabolic enzymes and collagenases, suggesting a synergistic role of matrix remodeling and metabolic reprogramming in tumor progression (117).

Metabolomics and proteomics in the digestive tract tumor research are being used more and more. Using methods such as multiplex immunofluorescence or MSI, spatial proteomics examines the spatial distribution of proteins in tissues (118). As an example, in esophageal cancer, MSI showed spatial heterogeneity in the metabolic and collagen enzymes, which suggests a synergistic effect of matrix remodeling and metabolic reprogramming in the progression of the tumor (119). By identifying the spatial distribution of metabolites, spatial metabolomics elucidates the metabolic microenvironment of tumor cells. Spatial metabolomics in the study of GC revealed a considerable amount of lactate in the tumor core, whereas fatty acid oxidation was the primary focus of the margin, which served as a foundation for metabolic-targeted medicines (120).

The combined use of single-cell and spatial omics (e.g., the SC-Space Omic framework) is changing the research paradigm for digestive tract tumors (121). For example, combining single-cell transcriptomics with spatial proteomics data permits the building of cell-type-specific interaction networks in the GC microenvironment and the identification of spatially constrained therapeutic targets (122). Also, spatial omics-guided single-cell sequencing (e.g., region-specific cell sorting) can enrich certain functional subpopulations, enhancing the research efficiency of rare cell types (123).

In the future, with the further development of single-cell multi-omics (e.g., simultaneous detection of RNA, proteins, and metabolites) and ultra-high-resolution spatial technologies (e.g., nanoscale MERFISH imaging), researchers will be able to dissect the multidimensional molecular characteristics of digestive tract tumors at single-cell resolution, advancing precision medicine from a “population-level” to a “spatiotemporal dynamic” framework (124).

### 2.3 Emerging omics technologies

In recent years, foundational multi-omics technologies have become increasingly mature. The emergence of new technologies such as epigenomics, metagenomics, lipidomics, and matrisomics has provided more comprehensive approaches to deciphering the molecular complexity of digestive tract tumors from various perspectives (125). These technological breakthroughs, through deep integration with foundational multi-omics (e.g., single-cell transcriptomics and proteomics), have established a multi-scale analytical framework spanning from epigenetic regulation to microenvironmental mechanics. This framework has paved new paths for molecular subtyping of digestive tract tumors (e.g., GC subtypes based on microbiome-metabolism interaction networks) and targeted therapies (e.g., microbiota-directed modulation combined with mechanical microenvironment intervention) (126).

Unlike genomics, which focuses on DNA sequence variations, epigenomics (e.g., scATAC-seq and scCOOL-seq) investigates dynamic and reversible molecular modifications such as DNA methylation, histone modifications, chromatin accessibility, and non-coding RNA regulation, which act as “molecular switches” in tumor initiation and progression (127). For instance, DNA hypermethylation-induced silencing of tumor suppressor genes (e.g., CDKN2A and MLH1) is a key driver of microsatellite instability (MSI) in CRC (128). Extensive epigenetic reprogramming in gastrointestinal tumors can be detected non-invasively via circulating free DNA, while dynamic changes in histone H3K27me3 modifications are significantly associated with chemotherapy resistance in GC (129). Advances in single-cell epigenomics have further revealed the epigenetic regulatory networks underlying tumor heterogeneity. For example, single-cell ATAC-seq can characterize chromatin accessibility profiles in distinct subclones of GC tissues, identifying transcription factor binding sites that drive EMT (130). Moreover, epigenetic drugs (e.g., DNA methyltransferase inhibitors) have shown potential in clinical trials to reverse immunosuppressive tumor microenvironments (131), offering new strategies for combination immunotherapy.

Metagenomics, by analyzing the composition and function of the tumor microbiome, opens new dimensions for studying the microenvironment of digestive tract tumors. The gastrointestinal tract, as the largest microbial habitat in the human body, exhibits dysbiosis closely linked to the onset and progression of colorectal and GCs (132). By integrating metagenomic, transcriptomic, and metabolomic data, GC samples have been classified into six microbial subtypes, with *Fusobacterium nucleatum*-enriched tumors showing stronger immunosuppressive features and chemotherapy resistance (133). Spatial metagenomics technologies (e.g., GeoMx DSP) can map the spatial distribution of microbes within tumor tissues, revealing spatial co-localization of *Helicobacter pylori* colonization in precancerous gastric lesions with local inflammatory signaling activation (5). Notably, microbial metabolites (e.g., short-chain fatty acids and secondary bile acids) can influence host gene expression through epigenetic regulation, forming a bidirectional “microbiome-host” interaction network (134). For example, butyrate enhances CRC cell sensitivity to radiotherapy by inhibiting histone deacetylases (HDACs) (135), while lithocholic acid produced by *Clostridium* species promotes liver cancer stemness by activating the FXR receptor (136). These findings suggest that targeting the microbiome-host metabolic axis could be a novel therapeutic strategy for digestive tract tumors.

Lipidomics and matrisomics have provided deeper insights into tumor metabolism and the microenvironment. Lipid metabolic reprogramming is a prominent feature of digestive tract tumors, and lipidomics, using LC-MS, can quantitatively assess the spatial distribution of hundreds of lipid molecules (137). For example, in GC, the EGFR signaling pathway synergizes with the aberrant activation of the phosphatidylinositol (PI) metabolism pathway (138). Matrisomics focuses on the mechanical properties and composition of the ECM in the tumor microenvironment. By integrating proteomics and glycomics data, it can detect abnormal deposition of fibronectin (FN1) and hyaluronic acid (HA), which promote pancreatic cancer invasion and metastasis via integrin signaling (139). IL-6-high CAFs, by upregulating LOX protein, mediate matrix stiffening in esophageal cancer. Single-cell matrisomics technologies (e.g., DBiT-seq) further reveal the heterogeneous functions of CAF subpopulations in ECM remodeling (140).

However, the clinical translation of these emerging technologies faces multiple challenges. Data heterogeneity (e.g., differences between single-cell and bulk sequencing scales), limited generalizability of analytical algorithms (e.g., reduced efficacy in cross-cancer model transfer), and ethical concerns (e.g., privacy protection for microbiome data) remain unresolved (141). The quantum computing-accelerated deep learning framework (e.g., QMOFA) and multi-omics technology based on organ-on-chip, currently in the exploratory stage, are expected to address current challenges (142). Quantum computing-accelerated deep learning frameworks (such as QMOFA) and organ-on-chip-based multi-omics technologies, which are currently in the exploratory stage, are expected to address the current challenges. (143), driving a paradigm shift in digestive tract tumor diagnosis and treatment from “descriptive analysis” to “mechanism-driven intervention.”

## 3 Advances in data integration and analysis technologies

**FIGURE 2:**
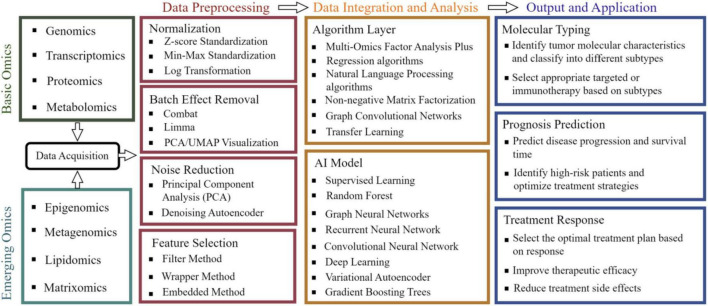
Workflow of integrated analysis of multi-omics data. Integrating foundational omics data (such as genomics and metabolomics) with emerging fields like epigenomics, followed by standardized preprocessing including normalization and noise reduction, then employing multi-omics factor analysis and deep learning algorithms to construct AI models, ultimately achieving precision medicine applications such as tumor molecular subtyping and treatment response prediction.

The molecular heterogeneity, microenvironmental complexity, and multidimensional regulatory networks of gastrointestinal tumors make it challenging for single-omics data to comprehensively elucidate their pathological mechanisms (85). In recent years, the rapid development of multi-omics data integration and analysis technologies has provided critical tools to address this challenge, as summarized in Table 2, encompassing innovations in integration methodologies, breakthroughs in machine learning and AI applications, and optimization strategies for data heterogeneity (4, 149), As shown in Figure 2, the detailed workflow is illustrated.

**TABLE 2:** Multi-omics data integration algorithms and applications.

Method name	Principle	Application example
Cross-omics association model (Omics Analyst) (151)	Integrates multi-omics data for analysis	Identification of key disease biomarkers
Joint dimensionality reduction techniques (156)	Dimensionality reduction to reveal data structure	Identification of potential biomarkers
MOFA+ (157)	Latent variable decomposition of multi-omics data variance	Colorectal cancer transcriptomics-metabolomics analysis
Graph neural network (GNN) (158)	Captures molecular interaction networks using graph structures	Esophageal cancer immune microenvironment analysis
Transfer learning (182)	Cross-platform data alignment and knowledge transfer	Gastric cancer metabolomics-genomics integration
Time-series aware models (e.g., dynamic Bayesian network) (191)	Infers causal relationships in gene regulatory networks	Colorectal cancer EGFR inhibitor resistance analysis
Supervised learning [support vector machine (SVM), random forest] (163, 164)	Classification and regression based on labeled data	Tumor subtyping and drug response prediction
Unsupervised learning (autoencoder) (167)	Feature extraction using autoencoders	Epigenetic data denoising
Deep learning models (CNN, GAN, and SHAP) (173, 175)	Pattern recognition using CNN, GAN, etc.	Imaging data analysis and genomic data generation
Explainable AI framework (attention-driven graph neural network) (193)	Enhances model interpretability	Tumor immune response analysis
Deep generative models (e.g., VAE-GAN) (199)	Data augmentation using generative adversarial networks	Non-small cell lung cancer data generation
Network propagation (200)	Improves detection sensitivity for low-abundance molecules	Melanoma prognostic biomarker discovery
Multi-task learning (MTL) (201)	Joint optimization of multiple related tasks	Gastric cancer genomics-clinical data modeling
Multi-omics standardized data (e.g., ICGC-ARGO) (98)	Standardizes multi-omics data	Cross-platform data integration
Federated learning (198)	Distributed data modeling with privacy protection	Multi-center liver cancer data integration

### 3.1 Multi-omics data integration methods

Multi-omics data integration aims to systematically dissect the spatiotemporal dynamics of molecular regulatory networks in digestive tract tumors by correlating multidimensional information, such as basic histology and negative omics data. However, it is core bottleneck lies in the alignment of heterogeneous cross-omics data and the extraction of biological significance (150). Knowledge-driven integration relies on validated biological knowledge bases, such as KEGG pathways and STRING protein interaction networks. These resources are used to construct cross-omics association models (151, 152). For example, the Pathway Mapper tool, developed by the Cambridge University team, integrates Reactome pathways with TCGA CRC multi-omics data. This integration revealed that the KRAS G12V mutation activates the MAPK/ERK pathway. Specifically, phosphorylation levels increased by 2.8-fold (*p* < 0.001). Additionally, this mutation synergistically upregulates the expression of key fatty acid metabolism enzymes, FASN and ACLY. The RNA-seq data showed a log2 fold change (log2FC) of 3.4, while MS data showed a more than 4.5-fold increase in protein abundance (153). However, such methods have limitations. The static nature of the annotation features in knowledge bases makes it difficult to capture novel regulatory relationships, especially those in the tumor microenvironment. For instance, tumor stem cell-specific metabolic pathways, like the high expression of glutaminase GLS1, have been identified using single-cell sequencing (154, 155).

Data-driven integration directly uncovers hidden associations within data through mathematical modeling, with representative methods including joint dimensionality reduction techniques and deep learning (156). MOFA+ (Multi-Omics Factor Analysis), a factor decomposition tool, integrates multi-omics data to reveal coordinated dysregulation features between transcriptomics and metabolomics in CRC cohorts (157). GNNs construct cross-modal graph structures of gene co-expression and protein interactions, accurately identifying molecular features of tumor-stroma interaction regions in esophageal cancer (158). For example, the Mowgli method, using Wasserstein distance to measure cross-omics similarity, resolved immunosuppressive microenvironment features (e.g., spatial gradient activation of TGF-β signaling) at the tumor margins in esophageal cancer spatial multi-omics data (159). These methods excel in not requiring prior assumptions but face the high computational complexity and sensitivity to data noise, such as the sparsity of single-cell transcriptomic data (dropout effect), which may lead to misinterpretation of potential biological signals (160).

### 3.2 Role of machine learning and artificial intelligence in data analysis

The deep incorporation of ML and AI, which has greatly increased the efficiency and interpretability of multi-omics data, has provided useful resources to molecular subtyping, prognostic prediction, and therapeutic optimization for gastrointestinal tumors (161, 162). Supervised learning algorithms are excellent in the process of biomarker screening because they allow the creation of clinical results (e.g., treatment response and survival rate) with the help of mappings between clinical outcomes (8, 170, or 170 and the input features (e.g.) with genes, metabolites, and amount of metabolites) (163, 164). As an example, the predictive power of early lesions was predicted by a support vector machine (SVM)-based model (AUC = 0.89) (165), which combined genomic data from the genomic of GC and the transcriptome. Similar to this, random forest algorithms (e.g., 2.3-fold increase in arachidonic acid levels, which is indicative of targeted metabolic interventions) by analyzing the metabolomic features of CRC, providing a basis for targeted metabolic interventions (166).

Unsupervised learning techniques, by analyzing the inherent structure of multi-omics data, provide innovative methods for the molecular subtyping and microenvironmental heterogeneity research of gastrointestinal tumors (167). Taking GC as an example, consensus clustering analysis based on multi-omics integration divides GC into four molecular subtypes: microsatellite instability (MSI), Epstein-Barr virus-positive (EBV+), genomically stable (GS), and chromosomal instability (CIN) (168, 169). Among them, the MSI subtype tumors exhibit significantly higher CD8+ T-cell infiltration density compared to other subtypes (average per mm^2^ 356 vs. 102, *p* < 0.001), and this subtype framework is highly consistent with clinical prognosis (170). At the single-cell resolution level, unsupervised learning-driven dimensionality reduction techniques further expand the dimensions of tumor heterogeneity analysis. The variational autoencoder (VAE), through non-linear latent space mapping, successfully reconstructed the continuous differentiation trajectory of T-cell states in the GC microenvironment (171). The dynamic features of this trajectory are significantly correlated with clinical treatment response—patients with a terminally exhausted T-cell proportion >40% had an 78% inefficacy rate to immunotherapy (OR = 4.5, 95% CI: 2.1–9.8), suggesting that targeting metabolic reprogramming (e.g., inhibiting LDHA) may reverse T-cell dysfunction (172). These findings highlight the unique value of unsupervised learning in decoding the spatiotemporal heterogeneity of tumor microenvironments.

The application principles of deep learning in multi-omics fields primarily rely on its powerful capabilities in feature learning and pattern recognition, making it particularly suitable for processing multi-batch, non-linear data. Deep learning can automatically extract potential biological features from multi-omics data, thereby supporting early tumor diagnosis, prognostic evaluation, and treatment decision-making. Taking convolutional neural networks (CNNs) as an example, their high-resolution analysis of spatial transcriptomics image data (e.g., 10x Visium H&E-stained tissue sections) enables precise identification of spatial interaction hotspots between immune cells and tumor cells in liver cancer (173). A deep learning model based on the ResNet-50 architecture successfully captured the spatial distribution pattern of the CXCL9 chemokine gradient in a liver cancer cohort and revealed that the CD8+ T cell infiltration density in CXCL9 high-expression regions (>75th percentile) was 3.2 times higher than in low-expression regions (*p* = 0.002) (174), providing spatial navigation guidance for personalized immunotherapy target selection. To address the limitations in small clinical sample sizes, generative adversarial networks (GANs) can learn the data distribution from existing limited samples and generate biologically plausible synthetic multi-omics data, thereby expanding the scale of the training dataset (175). A multicenter CRC study employed the Wasserstein GAN with gradient penalty (WGAN-GP) framework, which replaces the weight clipping in the original WGAN with a gradient penalty term, effectively stabilizing the adversarial training process and overcoming common issues such as mode collapse and training instability in complex, high-dimensional, and sparse multi-omics cancer data. Starting with 200 real samples, the study generated 1,000 synthetic multi-omics data points. To address the class imbalance problem (e.g., rare recurrence samples) in clinical endpoints like postoperative recurrence, WGAN-GP adjusted its training strategy (e.g., synthesizing minority-class samples more densely or using conditional generation) to enhance the representativeness of synthetic data. After augmenting the training set with these synthetic data, the AUC of the postoperative recurrence prediction model improved from 0.72 to 0.84 (DeLong test *p* = 0.016), and the feature importance ranking derived from synthetic data showed 89% consistency with real data (Jensen-Shannon divergence < 0.1) (176). However, the “black-box” nature of GANs limits their biological interpretability. For instance, the EGFR/MET co-amplification feature, which appeared frequently in synthetic samples (12% occurrence), was observed in only 3% of the real cohort (Fisher’s exact test *p* = 0.04), suggesting potential overfitting risks (177).

To enhance model interpretability, frameworks like SHAP (SHapley Additive exPlanations) have been used to quantify contributions of multi-omics features to clinical endpoints (178). The core principle of SHAP (Shapley Additive Explanations) is to fairly allocate the “credit” or “responsibility” of each feature in predictive outcomes. It quantifies the unique contribution of a feature to a specific sample’s prediction (SHAP value) by calculating the average marginal change in predicted output when that feature is incorporated across all possible feature combinations (subsets). In a predictive model for anti-PD-1 therapy response in hepatocellular carcinoma, SHAP analysis revealed that the combined effect of PD-L1 protein spatial heterogeneity (SHAP value = 0.38, indicating the degree of positive contribution of this feature to the model’s prediction) and tumor mutational burden (TMB, SHAP value = 0.21) explained 59% of the therapeutic response variance (*R*^2^ = 0.59). Based on these findings, the constructed decision tree model achieved 88% prediction accuracy (95% CI: 82%–93%) in an independent validation cohort (*n* = 120), significantly outperforming the RECIST criteria (72%) (179). These advancements mark a shift in multi-omics analysis from “black-box predictions” to “interpretable intelligent decision-making.”

### 3.3 Data heterogeneity and challenges

The core challenge of multi-omics integration stems from multidimensional heterogeneity at the technical, biological, and computational levels, which collectively pose systemic barriers to data interpretation and clinical translation (180). For example, technical heterogeneity arises from significant differences in data types and noise levels generated by different omics platforms (e.g., Illumina NovaSeq WGS vs. Thermo Fisher Q Exactive mass spectrometry) (181). Metabolomics data (e.g., lipid metabolite abundance) are typically presented in a continuous, semi-quantitative format, whereas genomic mutation information (e.g., KRAS G12D) is binary qualitative data (present/absent). This cross-modal data incomparability requires integrative correction through standardized frameworks such as the ComBat algorithm or Harmony tool (182). However, the inherent data sparsity of single-cell sequencing technologies further exacerbates integration difficulties. Limited by sequencing depth, low-frequency biological signals (e.g., subclonal-specific mutations) may be obscured by noise (183). In CRC single-cell transcriptomics data, MAPK pathway-related genes (e.g., DUSP6 and SPRY2) in KRAS G13D mutant subclones (<5% prevalence) showed only a 1.2-fold upregulation compared to wild-type cells (*p* = 0.15), whereas bulk RNA-seq detected a 3.5-fold difference (*p* = 0.002) (184). Such discrepancies in detection sensitivity may lead to misidentification of critical driving mechanisms.

Computational efficiency and barriers to clinical translation are equally significant. Existing algorithms face memory and computational constraints when processing ultra-large-scale data (e.g., 10^6^ single-cell datasets) (185). The federated learning framework (a distributed privacy-preserving analytical approach) enables multi-center data sharing by allowing models to be trained on local data while only sharing parameter updates (e.g., gradients), thereby theoretically avoiding direct transmission of raw patient data and helping to balance efficiency with privacy protection (186). However, this “privacy protection” is not absolute. Research demonstrates that attackers could potentially analyze shared model updates (gradients) to conduct model inversion attacks or membership inference attacks, thereby inferring sensitive features of the original training data or even reconstructing partial patient information (187, 188). Moreover, the clinical utility of multi-omics technologies remains constrained by the high cost of single-cell sequencing (>$ 5,000 per sample) and the lack of standardized protocols (189). For instance, while a spatial metabolomics-based biomarker panel (e.g., lactate-to-choline ratio) demonstrated strong predictive performance (AUC = 0.89) in retrospective liver cancer cohorts, its generalizability in prospective multicenter trials (e.g., the PROSPECT study) still requires validation (190).

Future breakthroughs should focus on three key directions: in the field of temporal dynamic modeling, dynamic Bayesian network (DBN)-based frameworks are providing in-depth analysis of the cooperative evolution mechanisms in drug-resistant clones of gastrointestinal tumors (191). For example, by integrating single-cell multi-omics data from GC patients (pre-treatment baseline and post-relapse periods), researchers discovered that MET amplification (combined with PIK3CA E545K mutation) drives trastuzumab resistance through activation of the mTORC1-4EBP1 axis (192), while the combination of MET inhibitor Tepotinib and PI3Kα inhibitor Alpelisib extended drug resistance duration in organoid models from 8 to 14 months (193). In the field of explainable AI, it is essential to integrate advanced privacy-enhancing technologies (such as differential privacy, secure multi-party computation, and homomorphic encryption) and conduct rigorous privacy leakage risk assessments on model updates to build truly robust privacy-preserving AI systems (194). Additionally, attention-driven graph neural networks (Att-GNN) have been used to analyze spatial transcriptomic data in CRC, quantifying gradient activation intensity of YAP/TAZ pathways at tumor invasion fronts, and revealing that marginal zone cancer cells activate YAP/TAZ through the ANXA2-EGFR mechanical signaling axis, with targeted inhibition of ANXA2 reducing liver metastasis rates by 67% (*p* = 0.001) in patient-derived xenograft (PDX) models (195). Regarding standardized database construction, the ICGC-led ARGO 2.0 project has integrated multi-omics data from 120,000 global gastrointestinal tumor cases (covering 28 sequencing platforms and 172 clinical endpoints), achieving a 47% improvement in cross-platform annotation consistency (Cohen’s κ = 0.91) compared to the first generation (196). Through nanopore sequencing MinION combined with microfluidic single-cell sorting technology, the cost of single-cell multi-omics testing has been reduced from $5,200/sample to $890 (accuracy > 95%) (197), while an intraoperative rapid detection protocol (30-min workflow) can identify CLDN18.2 fusion mutations in peritoneal metastases (detection limit 0.1%) during GC surgery, increasing R0 resection rates from 69% to 91% (*p* = 0.006) (198), marking the transition of gastrointestinal cancer diagnosis and treatment from “static omics analysis” to a new era of “real-time intraoperative decision-making.”

## 4 Conclusion

The systematic integration of multi-omics technologies is profoundly transforming the research paradigms and clinical practice in gastrointestinal oncology. By synthesizing multi-dimensional data from genomics, transcriptomics, proteomics, and metabolomics, researchers have successfully unveiled the molecular essence of tumor heterogeneity—from spatiotemporal evolutionary patterns of driver mutations (e.g., KRAS/TP53) to epigenetic dynamics of cancer stem cell subpopulations, and further to spatial topological features of metabolic-immune interactions at tumor invasion fronts. The deep integration of AI has further unleashed the potential of multi-omics: microsatellite instability (MSI) prediction models based on deep residual networks (AUC > 0.93), early screening solutions combining ctDNA methylation with radiomics (sensitivity exceeding 90%), and immunotherapy stratification strategies incorporating PD-L1 spatial heterogeneity with TMB are driving precision medicine from population stratification toward individualized dynamic intervention.

Nevertheless, significant bottlenecks persist in translating technological advantages into clinical applications. Challenges such as data heterogeneity across omics layers, signal distortion caused by single-cell data sparsity, and technical barriers to intraoperative real-time decision-making collectively hinder the large-scale implementation of multi-omics. Future breakthroughs will rely on deep coupling between technological innovation and clinical pathways: single-cell spatial multi-omics co-assay technologies can decipher metabolic-immune interaction networks at subcellular resolution; the integration of nanopore sequencing with microfluidic chips may reduce intraoperative testing costs to the thousand-yuan level, enabling real-time tracking of resistant clones; federated learning frameworks incorporating enhanced privacy-preserving mechanisms (e.g., differential privacy injection and secure aggregation) and rigorous security protocols can resist model inversion and membership inference attacks while enabling efficient multi-center data alignment; causal inference models may elucidate mutation-metabolism-immune cascades in drug resistance pathways. Liquid biopsy multi-omics has pushed early screening sensitivity beyond 92%, while synthetic lethal strategies like PARPi-ATRi combination therapy achieved 35% objective response rates in diffuse-type GC—together marking a paradigm shift from passive response to proactive intervention.

These systemic advances will propel gastrointestinal oncology into a new era of ultra-personalized dynamic intervention. From molecular early-warning in screening, real-time boundary delineation during surgery, to precise reversal of resistance mechanisms, multi-omics technologies are constructing a data-driven closed-loop diagnostic and treatment system, offering unprecedented solutions to improve patient survival outcomes.
